# Cyclodextrin-mediated Enhancement of Haloperidol Solubility: Physicochemical Studies and *In Vivo* Investigation Using Planaria Worms

**DOI:** 10.1007/s11095-025-03909-0

**Published:** 2025-08-15

**Authors:** Yuehuai Xiong, Kenneth Shankland, Vitaliy V. Khutoryanskiy

**Affiliations:** 1 Reading School of Pharmacy, University of Reading, Whiteknights, PO Box 224, Reading, RG66AD UK; 2 Physicochemical, Ex Vivo and Invertebrate Tests and Analysis Centre (PEVITAC), University of Reading, Reading, RG66DX UK

**Keywords:** Cyclodextrins, Haloperidol, *in vivo* model, Inclusion complex, Invertebrates, Planaria

## Abstract

**Purpose:**

This study aims to evaluate the ability of various cyclodextrins (CDs) to enhance the aqueous solubility of haloperidol (HAL), through the formation of inclusion complexes. It also investigates the pharmacological activity of CD/HAL complexes using a planaria model.

**Methods:**

Inclusion complexes were prepared using α-CD, β-CD, methyl-β-CD, hydroxypropyl-β-CD and γ-CD. The solubility of HAL in the presence of CDs was assessed, and the stoichiometry of the complexes was determined using Job’s method. Physicochemical interactions between HAL and CDs were characterized by nuclear magnetic resonance (NMR), differential scanning calorimetry (DSC), and powder X-ray diffraction (PXRD). The *in vivo* pharmacological activity was tested in planaria worms following exposure to HAL in the presence or absence of CDs.

**Results:**

HAL’s aqueous solubility was significantly enhanced in the presence of α-CD, methyl-β-CD, and hydroxypropyl-β-CD, while γ-CD showed no effect. Only modest solubility improvements were observed with increasing β-CD concentrations up to 8 mg/mL. Stoichiometric analysis confirmed a 1:1 ratio of HAL to CD in the inclusion complexes. *In vivo* studies demonstrated that HAL reduced planaria mobility, mimicking cataleptic effects seen in mammals, whereas the presence of CDs reduced this pharmacological effect.

**Conclusion:**

Cyclodextrins, particularly α-CD, methyl-β-CD, and hydroxypropyl-β-CD, effectively enhance the solubility of haloperidol by forming 1:1 inclusion complexes. The reduction in haloperidol-induced behavioral changes in planaria by CDs suggests a potential impact on drug bioavailability and supports the use of planaria as a simple *in vivo* model for screening neuroactive compounds and formulations.

**Supplementary Information:**

The online version contains supplementary material available at 10.1007/s11095-025-03909-0.

## Introduction

Cyclodextrins (CDs), initially described in 1891, are a family of cyclic oligosaccharides primarily composed of six (α-cyclodextrin), seven (β-cyclodextrin), eight (γ-cyclodextrin), or more glucopyranose units, interconnected through α-(1,4) bonds [1]. In 1911, they were successfully crystallized and designated as'crystallised dextrin α'and'crystallised dextrin β.'Subsequently, various novel synthesis techniques and methodologies were devised to fulfill the laboratory-scale demand for producing cyclodextrins. Since then, CDs have garnered significant attention from scholars, leading to a surge in scientific publications and patent applications, showcasing their diverse applications as specialty chemicals and indispensable components in chromatographic methods, stabilizers, catalysts, etc [2]. The principal attribute of CDs hinges on their capacity to'encapsulate'molecules at the molecular level. Over the past few decades, there has been an ever-expanding number of applications of CDs in numerous domains, including the food science [3], chemical compound separation [4], pollutant removal [5], and drug formulation. Solubility and permeability enhancement are critical and widely popular topics in pharmaceutics [6]. Cyclodextrins are well-known for their ability to enhance both properties, making them valuable excipients in drug formulation [7].

Haloperidol (HAL) is an antipsychotic compound with a primary application in the management of conditions such as schizophrenia, delusions, and hallucinations [8], HAL is a weakly-basic BCS Class II substance with p*K*_a_ 8.65 (Drug Bank) [9], a low aqueous solubility of less than 0.1 mg/mL and oral bioavailability *ca*. 60–65% [10]. HAL is commonly used as a model drug due to its poor aqueous solubility.

In the pursuit of enhancing the solubility and bioavailability of haloperidol, commonly available cyclodextrins (CDs), including α-, β-, and γ-CD, have been used as host molecules. Cyclodextrin inclusion complexation relies on the establishment of host–guest inclusion complexes through weak intermolecular interactions, a technique widely employed to address the solubility challenges associated with poorly water-soluble drugs [11]. Despite the wide use of α- and β-CD, along with their derivatives, for solubility enhancement, it is important to note that α- and β-CD themselves possess relatively low aqueous solubilities of 15 mg/mL and 18 mg/mL, respectively [12]. As such, in this study, hydroxypropyl β-CD (HP-β-CD) and methyl β-CD (Me-β-CD) were selected as agents to significantly enhance the solubility of HAL in ultrapure water due to their higher aqueous solubilities of 65 mg/mL and 60 mg/mL, respectively.

The association of haloperidol with cyclodextrins has been investigated in several previous studies. Pisaniello *et al *[13] examined the complexation of haloperidol with α- and γ-cyclodextrins using ^19^F NMR spectroscopy by monitoring chemical shift changes in the fluorine nucleus of the drug. They estimated the stability constants and concluded that the values are relatively low, suggesting that these cyclodextrins may not be effective carriers for haloperidol in clinical drug delivery applications. Loukas [14] employed ^1^H NMR spectroscopy to study the interaction between haloperidol and hydroxypropyl-β-cyclodextrin, confirming the formation of 1:1 inclusion complex. In a different study, Loukas *et al *[15] investigated the complexation of haloperidol with both methyl-β-cyclodextrin and hydroxypropyl-β-cyclodextrin. They observed a 20-fold and 12-fold increase in drug solubility with a 10-fold excess of methyl-β-cyclodextrin and hydroxypropyl-β-cyclodextrin, respectively, and confirmed a 1:1 stoichiometry for both inclusion complexes. More recently, Omari *et al *[16] investigated the complexation of haloperidol with β-cyclodextrin using a comprehensive set of techniques, including phase solubility analysis, 1H NMR spectroscopy, X-ray powder diffraction, differential scanning calorimetry, scanning electron microscopy and molecular mechanical modeling. They concluded that both protonated and neutral haloperidol form soluble 1:1 and 1:2 (haloperidol: β-cyclodextrin) inclusion complexes, although ionization of the drug significantly reduces its tendency to form such complexes.

Haloperidol exerts a characteristic effect in rodents, inducing a state of catalepsy—a transient condition marked by immobility, diminished responsiveness to external stimuli, and the maintenance of abnormal postures for prolonged periods. This response is widely employed as a behavioral model to investigate haloperidol’s impact on dopaminergic pathways and to assess its extrapyramidal side effects [17].

To the best of our knowledge, no previous studies have examined the effect of haloperidol on lower animals such as planaria. These invertebrate flatworms possess a relatively simple yet functionally relevant nervous system, including dopaminergic signaling pathways, making them promising candidates for neuropharmacological investigations. Employing live invertebrates such as planaria to study the pharmacological effects of haloperidol and its formulations offers a valuable opportunity to reduce dependence on mammalian models. This strategy aligns with the 3Rs principles—Replacement, Reduction, and Refinement—by facilitating more ethical and sustainable approaches to early-stage drug testing [18].

In the present study, the effects of various cyclodextrins (α-, β-, γ-, hydroxypropyl-β-cyclodextrin, and methyl-β-cyclodextrin) on the aqueous solubility of haloperidol were evaluated. The interactions between haloperidol and cyclodextrins in solution were comprehensively investigated using proton nuclear magnetic resonance (NMR) spectroscopy, and the stoichiometry of the resulting inclusion complexes was determined spectrophotometrically via Job's plot analysis. The crystallinity of haloperidol within the solid-state inclusion complexes was assessed using differential scanning calorimetry (DSC) and powder X-ray diffraction (PXRD). The pharmacological effects of free haloperidol and its inclusion complexes were then examined *in vivo* using *Schmidtea mediterranea* planarians.

## Materials

Haloperidol, α-, β-, γ-, HP-β-CD and Me-β-CD (BioReagent, ≥ 97%), methanol, hydrochloric acid, sodium dihydrogen phosphate, monopotassium phosphate, deuterium oxide and acetic acid-d were all purchased from Scientific Laboratory Supplies (UK) and used without further purification.

## Methods

### Spectrophotometric Determination Method of Hal

HAL quantification was carried out utilizing a UV–Visible spectrometer (Jenway 7315). The analysis was carried out at a detection wavelength of 246 nm, employing a 1 mL sample volume. Calibration curves were established using a solvent blend consisting of methanol, ultrapure water, and concentrated hydrochloric acid in a volumetric ratio of 49:50:1 (v/v/v). To prepare these calibration solutions, 10 mg of HAL was dissolved in this solvent mixture in a 25 mL volumetric flask. Subsequently, a stepwise dilution process was used, resulting in a range of solutions of varying concentration:1.25 μg/mL, 2.5 μg/mL, 5 μg/mL, 10 μg/mL, 20 μg/mL, and 30 μg/mL.

### Phase Solubility Diagram

An excess amount of HAL (0.5 mg/mL) was mixed in pure water containing increasing amounts of CD using a magnetic stirrer at room temperature for 72 h. In order to separate excess insoluble HAL, samples were filtered through 0.45 µm syringe filters. The concentration of HAL in each aqueous solution was spectrophotometrically determined at 246 nm. Experiments were carried out in triplicate at every sample point.

Experimental results were analyzed statistically using Student’s t-test as implemented in GraphPad Prism 8.0.2 software (GraphPad, Boston, Dotmatics company, USA). Each maximum value of solubility of CDs was compared with intrinsic solubility of haloperidol via statistical analysis using p < 0.05 as a significance cut-off.

### Determination of Inclusion Complex Stoichiometry

The formation of inclusion complexes between HP-β-CD, Me-β-CD, and α-CD with HAL in aqueous solution was analyzed using the Job’s plot method. The difference between absorbance intensity with and without CD (ΔA) at 246 nm was determined using the mixtures of HAL:CD having different molar ratios (from 0 to 1) but keeping the total molar concentration of the components constant. The mixtures were prepared using equimolar solutions of HAL and CD. ΔA × R was plotted against R, where R is defined by the following equation:

$$R=\frac{Fraction\;of\;HAL}{Fraction\;of\;(CD+HAL)}$$

There are some conditions that must be met in order that Job’s method is applicable. The property being studied must vary in direct proportion to the concentration of the components. More importantly, there must only be one complex in solution which predominates over all others under the conditions of the experiment [19]. In this experiment, inclusion complexes of HAL and CD meet the requirements of Job’s plot.

### ^1^H NMR Spectroscopy

Haloperidol solution with 0.1 mg/mL was prepared in D_2_O containing 2% acetic acid-d to increase its solubility to meet the requirement of NMR detection limit. Then, an equal molar concentration of HP-β-CD was added to half of the solution to form inclusion complexes. After that, the samples of HAL and HAL/HP-β-CD in D_2_O were filtered through 0.22 μm filters and then their ^1^H NMR spectra were recorded with a Bruker DRX Avance 600 spectrometer. The data was processed with Mestrenova S.L software (Mestrelab Research, Barcelona, SPAIN).

### DSC

Thermal analysis was conducted on pure HAL, the cyclodextrins, and their inclusion complexes using DSC Q2000 (TA Instruments, UK). For the DSC analysis, HAL, CD, and HAL/CD powders were prepared. Excess HAL and CD powders (50 mg/mL) were mixed with ultrapure water and stirred for 3 days, respectively. Subsequently, the solution was freeze-dried, yielding powdered samples. These powdered samples, weighing between 3–5 mg, were then carefully loaded into pierced Tzero aluminum pans. The thermal behavior of each sample was examined under a nitrogen atmosphere, employing a heating/cooling rate of 10 °C/min. In this experimental phase, several methods (ball milling, grinding, and whirl mixing) were employed for the preparation of physical mixtures. Each method involved combining 20 mg of HAL and 12.5 g of CD within their respective preparation devices. These mixture powders were then subjected to analysis via DSC as outlined above.

### PXRD

The samples for PXRD analysis were prepared using the same method as employed in the DSC experiment. Approximately 10 mg of each dry sample containing HAL, α-CD, HP-β-CD, ME-β-CD, HAL/α-CD, HAL/HP-β-CD and HAL/ME-β-CD was carefully placed onto a zero-background silicon plate. These samples were then measured using a Bruker D8 ADVANCE diffractometer equipped with Cu Kα_1_ radiation (40 mV, 40 mA; λ = 1.54056 Å) over the range 5–64° 2θ. A step size of 0.05° and a count time of 1.2 s per step were employed during the analysis process. Additionally, the sample was rotated at a speed of 30 rpm to help improve powder averaging.

### *In Vivo *Experiments

*Schmidtea mediterranea* planaria were kindly provided by Dr Jordi Solana (Oxford Brooks University) and bred at room temperature in 10 L plastic containers containing artificial pond water (APW) and fed weekly with calf liver. Worms were fasted for at least 5 days prior to testing. APW was prepared by mixing 5.0 M NaCl (3.2 mL), 1.0 M CaCl_2_⋅6H_2_O (10.0 mL), 1.0 M MgSO_4_ (10.0 mL), 1.0 M MgCl_2_ (1.0 mL), and 1.0 M KCl (1.0 mL). To this mixture, 1.008 g of NaH_2_CO₃ was added, then diluted with ultrapure water to a final volume of 10 L.

All experiments were conducted in glass petri dishes with 50 mL of sample media including pure HAL, HAL/α-CD, HAL/β-CD, HAL/HP-β-CD, HAL/Me-β-CD and control group. In all sample groups, HAL was tested at concentrations of 1.75 μg/mL, 3.5 μg/mL, 7 μg/mL, and 14 μg/mL, with consistent concentration of 14 mg/mL of all CDs added and forming inclusion complexes with HAL. Planaria were placed in a media-filled glass petri dish which was overlaid upon graph paper to allow the estimation of the distance over which the planaria moved. Using graph paper as a visual guide, the cumulative lines of planaria crossings were systematically tracked over the duration of a video recording. A coordinate system was established correlating time with the cumulative number of lines observed. Subsequently, the experimental data underwent statistical analysis employing Student’s t-test using GraphPad Prism. Each sample was compared against the control group, pure HAL, via statistical evaluation. A significance level of p < 0.05 was used.

## Results and Discussion

### Effect of Cyclodextrins on Aqueous Solubility of Hal

**Fig. 1:**
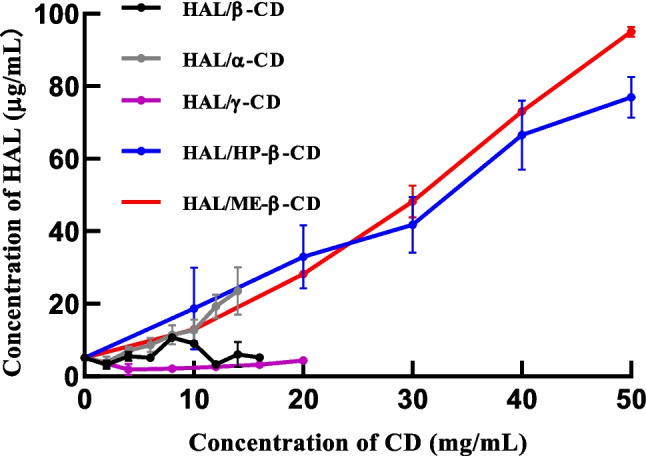
The effect of the concentration of various CDs on the aqueous solubility of HAL at room temperature.

Solubility studies were performed according to the method of Higuchi and Connors, which monitors the increase in a drug solubility in the presence of increasing concentrations of CD [16]. The UV calibration curve, displayed in Supplementary Information Fig. 1, meets the necessary requirements for accurate determination. As the inclusion complex itself is more soluble than HAL, the overall result is an increase in HAL solubility. Phase solubility studies were carried out in aqueous systems. The effect of CD concentration on the aqueous solubility of haloperidol was studied at room temperature and the results are shown in Fig. 1.

Firstly, the intrinsic solubility of HAL in ultrapure water is 5.1 ± 0.2 μg/mL, which is larger than 2.5 μg/mL reported previously [20]. Furthermore, the plot shows that HP-β-CD, Me-β-CD and α-CD improve the intrinsic solubility of HAL in ultrapure water whereas γ-CD does not enhance it. The relationship between HAL solubility and β-CD is more complex: solubility initially increases with rising β-CD concentration, reaching a maximum at 8 mg/mL, after which further increases in β-CD concentration lead to a decrease in HAL solubility. Due to their greater solubility in water (approximately 65 mg/mL and 60 mg/mL, respectively), HP-β-CD and Me-β-CD that included HAL were able to achieve the highest solubilities of 77 ± 5 μg/mL and 97 ± 2 μg/mL, respectively. It appears that the solubilizing effect of Me-β-CD is significantly (p < 0.05) greater than that of HP-β-CD at the concentration of 50 mg/mL. This could be attributed to the higher aqueous solubility of the methylated derivative and or a slightly better complexing ability of the derivative. Compared with the solubilizing power of HP-β-CD and Me-β-CD, when the concentration of α-CD was 14 mg/mL, the solubility of HAL increased to 23.0 ± 6.0 μg/mL, approximately 5 times greater than the intrinsic one (p < 0.05). γ-CD failed to increase the solubility of HAL. HP-β-CD and Me-β-CD are capable of loading significantly more HAL, as confirmed by the statistically significant differences in solubility. It has been reported in literature that the solubility of haloperidol can be improved to 8 μg/mL at a β-CD concentration of 6 mg/mL, an outcome that is in line with our results [16]. The solubility of haloperidol reached its maximum of 10.7 ± 0.6 μg/mL at a β-CD concentration of 8 mg/mL. In addition, in the presence of γ-CD the solubility of HAL was even lower (3.6 ± 0.5 μg/mL) when the concentration of γ-CD was 4 mg/mL.

The stability constant (*K*_st_) is commonly used in the literature to describe the binding strength and equilibrium between the components of the inclusion complex formed [16]. This constant can be calculated using the following equation, provided that the relationship between drug solubility and cyclodextrin concentration is linear:

$${K}_{St}=\frac{\text{s}lope}{{\text{S}}_{0}*(1-\text{slope})}$$

where S_0_ represents the intrinsic solubility (in mol/L) of HAL in ultrapure water, and the slope refers to the gradient of the linear portion of the graph.

The experimental dependence of drug solubility on cyclodextrin concentration was nearly linear for HP-β-CD/HAL but less so for Me-β-CD/HAL. Slope values were calculated by linear regression using all the HP-β-CD/HAL data points, and using the four data points that constituted the linear part of the Me-β-CD/HAL plot. The *K*_st_ values obtained were 439.5 M^−1^ and 590.6 M^−1^ for HP-β-CD/HAL and Me-β-CD/HAL, respectively.

To evaluate the significant disparities between the solubility of inclusion complexes formed with each type of CDs and the pure HAL, a Student t-test was employed. For this analysis, the maximum solubility value attained by each inclusion complex was used as the basis for comparison. Significant differences in the mean solubilities were tested at 95% confidence. The solubilities of haloperidol in the presence of α-CD, β-CD, Me-β-CD and HP-β-CD (14 mg/mL, 8 mg/mL, 50 mg/mL, 50 mg/mL, respectively) are significantly higher (p < 0.0001) when compared to the solubility of pure haloperidol. Furthermore, there was also a significant difference even at 10 mg/mL of Me-β-CD or HP-β-CD, which indicated that Me-β-CD and HP-β-CD improve solubility of haloperidol. In addition, the solubilizing capabilities of Me-β-CD and HP-β-CD were also compared, which demonstrated that the enhancement of solubility in the presence of Me-β-CD was significantly better compared to HP-β-CD (p < 0.0001). This means that Me-β-CD has the best solubilizing capability to haloperidol among these four CDs. Although the solubility of HAL/β-CD was significantly different to the solubility of pure haloperidol, an improvement in the drug solubility was only a factor of two.

### Determination of Inclusion Complexes Stoichiometry

**Fig. 2:**
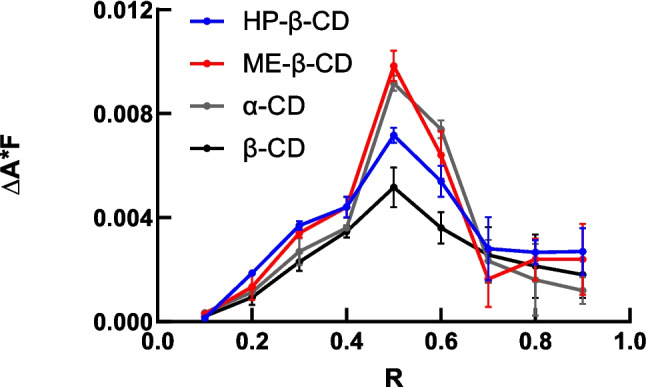
Job’s plot chart for inclusion complexation between unprotonated HAL and CDs. The stoichiometry of all CDs with HAL is 1:1.

Inclusion complex formation of HAL with different CDs in aqueous solution was characterized by UV spectrophotometry. The stoichiometry of the complexes formed between HAL and CDs was determined using Job’s method [21]. Peaks of Job’s plot identify the ratio of guest molecules to host molecules at stoichiometric proportions and thus allows one to determine the composition of the inclusion complex. Several Job’s plots are shown in Fig. 2. A maximum at R = 0.5 indicates that the stoichiometry of inclusion complexes is CD:HAL (1:1).

There are no reports about the Job’s plot of CDs and unprotonated HAL determined using UV spectrophotometry in the literature. However, there are reports for complexes of cyclodextrins with protonated haloperidol. For example, the chemical shift of NMR was used to build a Job’s plot for HP-β-CD and protonated HAL [14], in which the stoichiometry of CD and HAL was also 1:1. The stoichiometry of 1:1 was also verified in some other studies [16]. However, in molecular docking studies, both stoichiometries of 1:1 and 1:2 were predicted using Maestro 9.2 software (Schrodinger Inc.) [22]. In other literature using binding energy calculations, the piperidine and fluorophenyl moieties of HAL were shown to form optimal 1:2 complexes, which was predicted to be most stable [16]. In this study, the stoichiometry result is consistent with the 1:1 composition determined experimentally by others [20].

### ^1^H NMR Spectroscopy Study

^1^H NMR spectroscopy is a powerful technique for elucidating the structure of inclusion complexes formed by cyclodextrins in solutions, offering insights into the interactions between the cyclodextrin and the guest molecule [23].

**Fig. 3:**
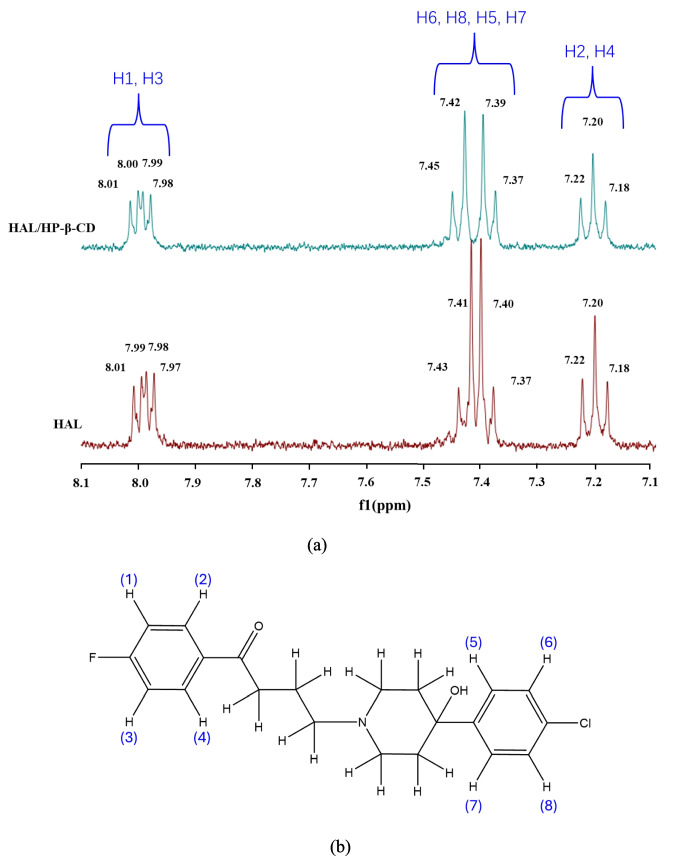
Aromatic region of the ^1^H NMR spectrum of haloperidol and its inclusion complex with HP-β-CD, recorded in D_2_O solutions with addition of CD_3_COOD (**a**); Chemical structure of haloperidol with aromatic protons labelled according to their assignment in the.^1^H NMR chemical shifts (**b**).

In this study, the ^1^H NMR spectra of HAL, HP-β-CD, and their inclusion complex were all recorded in deuterium oxide with addition of deuterated acetic acid (CD_3_COOD) to improve solubility of haloperidol (Fig. 3).

The aromatic region of the ^1^H NMR spectrum of haloperidol undergoes subtle but meaningful changes upon complexation with HP-β-CD. Compared to the spectrum of free haloperidol, where signals appear at 8.01, 7.99, 7.98, 7.97, 7.43, 7.41, 7.40, 7.37, 7.22, 7.20 and 7.18 ppm, the inclusion complex exhibits similar chemical shifts but with slight upfield shifts and splitting perturbations. The most pronounced shifts are observed for the signals corresponding to protons H5, H6, H7 and H8, which are located on the 4-chlorophenyl ring. The assignments for these peaks were taken from Loukas *et al *[15]. Protons H2 and H4 show no chemical shift changes, and only minor variations are observed for H1 and H3; all are associated with the 4-fluophenyl ring. The observed chemical shift changes suggest that the 4-chloriphenyl ring is likely included within the HP-β-CD cavity, whereas 4-fluorophenyl ring appears to be more exposed to the aqueous environment. These results are consistent with the 1H NMR study of similar complexes reported by Loukas *et al *[15]. However, in our case, the spectra exhibit significantly improved signal resolution. The data support the formation of 1:1 inclusion complexes, in which only one aromatic ring of haloperidol—the 4-chlorophenyl ring—is involved in inclusion complexation.

### DSC

**Fig. 4:**
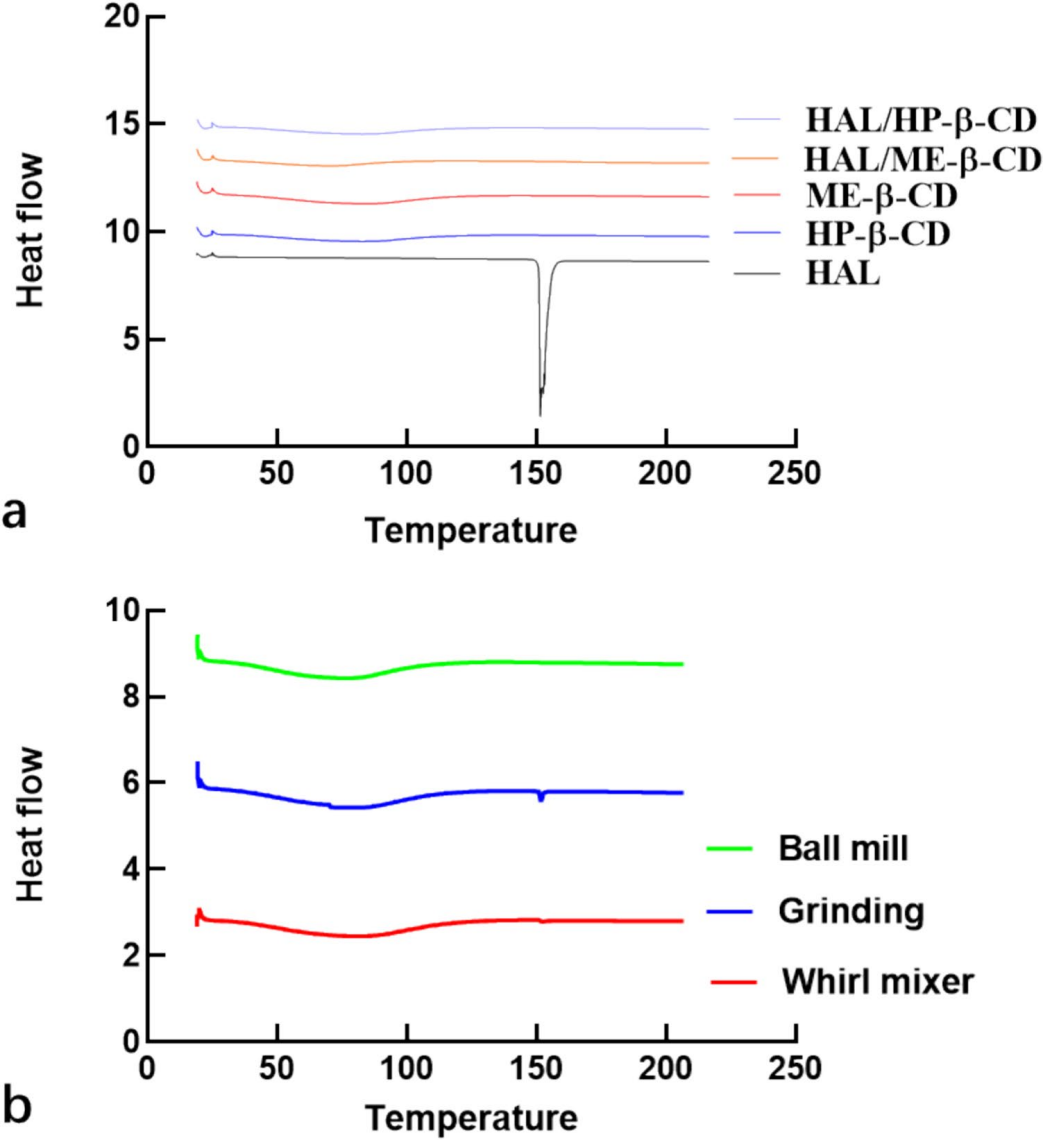
DSC thermograms of HAL, HP-β-CD, ME-β-CD as well as the freeze-dried inclusion complexes HAL/HP-β-CD, and HAL/ME-β-CD (**a**); different physical mixtures (**b**).

To study the thermal behavior of inclusion complexes and any differences to that of pure HAL, a DSC experiment was conducted using solid HAL, freeze-dried inclusion complexes, as well as drug-CD physical mixtures. The DSC curves of HAL, HP-β-CD, HAL/HP-β-CD, ME-β-CD, HAL/ME-β-CD and 3 kinds of physical mixtures are shown in Fig. 4. HAL displayed one sharp endotherm at 150 ℃, which is the melting point of its crystalline form [24]. A broad endotherm was observed at approximately 80 ℃ in the thermograms of HP-β-CD and Me-β-CD, which is likely due to the loss of physically-bound water; these results were consistent with a previously published study [25]. The sharp HAL endotherm is absent in both inclusion complexes, indicating that the inclusion complexes prepared by means of freeze-drying process are amorphous.

Next, physical mixtures of HAL and CDs were prepared by three different methods: whirl mixing, grinding and ball milling. These methods were used to ensure that the ingredients were evenly mixed, without applying conditions specifically intended to promote inclusion complex formation. Whirl mixing involves gentle agitation to blend the two materials together without applying significant mechanical force. Grinding in a mortar and pestle is a mechanical process that applies moderate pressure and shear to reduce particle size and mix components. Ball milling subjects the powders to higher-energy collisions between stainless steel balls contained in an oscillating chamber, leading to finer particle sizes and more intimate mixing. These methods vary in their intensity and level of energy input (ball milling > grinding > whirl mixing), which can influence the homogeneity of the mixture and the likelihood of interaction between components. As can be seen from Fig. 4b, three kinds of physical mixtures show a small peak at 150 ℃ corresponding to the melting point of HAL. Compared with inclusion complexes, all of the physical mixtures have this characteristic melting peak of HAL and the disappearance of the drug melting peak in inclusion complexes suggests that it is incorporated in the cavities of CD in a fully amorphous form. All the melting peaks in different kinds of physical mixture were integrated by software and degree of crystallinity was calculated using the equation below:

$$\mathrm{Degree}\;\mathrm{of}\;\mathrm{crystallinity}=\frac{\int physical\;mixture}{\int crystalline\;HAL}\times100\%$$,

where $\int physical \,mixture$ represents the area under the melting peak for a physical mixture, and $\int crystalline\, HAL$ represents the area under the melting peak for the crystalline HAL powder. The degree of HAL crystallinity in the physical mixtures calculated by the peak integration of samples prepared by whirl mixer, grinding and ball milling were 99%, 99%, and 0.9%, respectively. HAL crystallinity is retained in formulation prepared by whirl mixer and grinding but lost in ball mill sample.

### PXRD

**Fig. 5:**
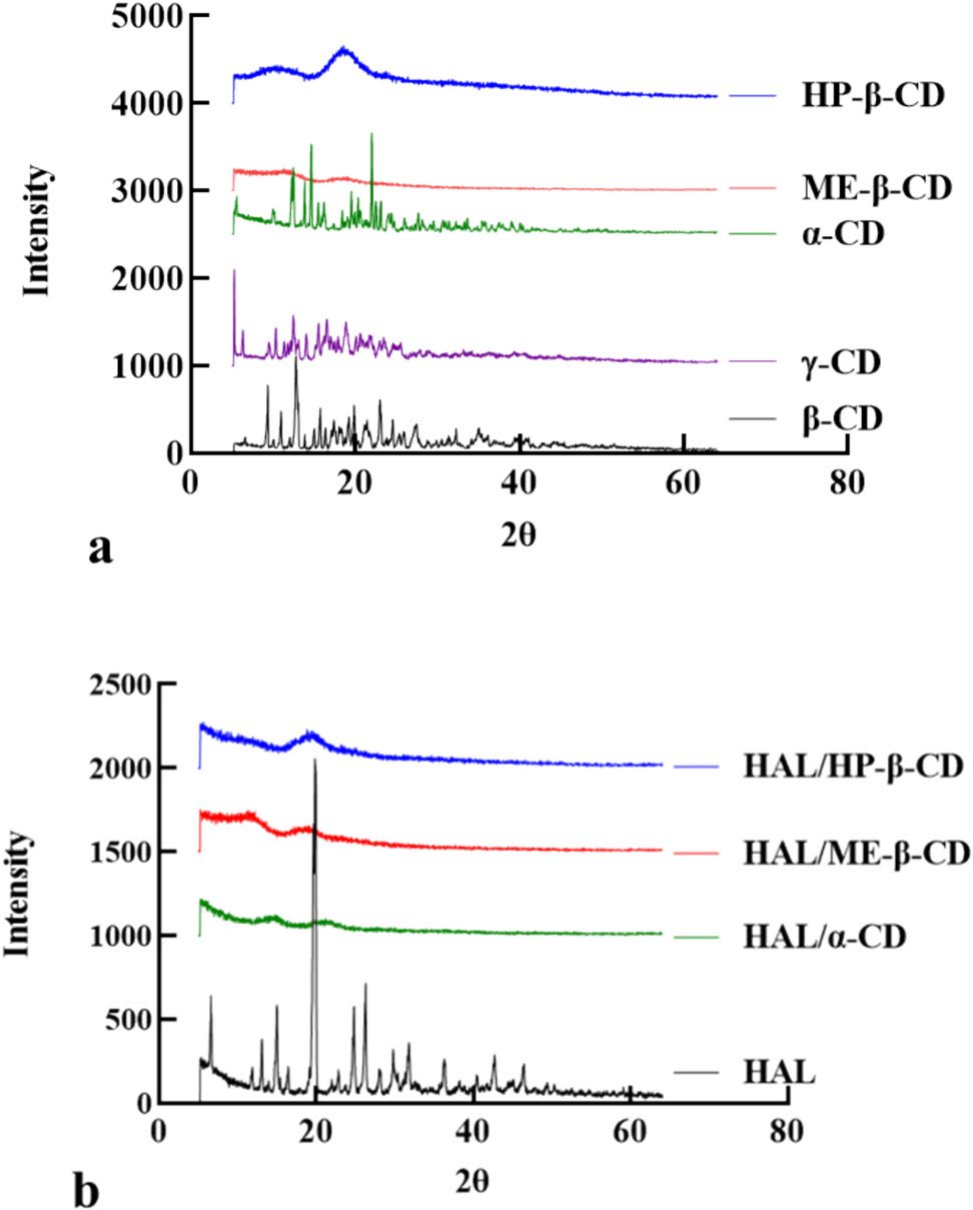
PXRD patterns of 5 kinds of CD (**a**); HAL and inclusion complexes (**b**).

PXRD was employed to evaluate the crystalline properties of the inclusion complexes in the solid state. Figure 5 shows PXRD patterns for the individual components, including HAL, CDs, and the HAL/CDs inclusion complexes. α-CD, β-CD and γ-CD show the sharp diffraction peaks indicative of crystalline materials; such peaks are absent in the much-less crystalline HP-β-CD and ME-β-CD samples. For example, the diffraction pattern of HP-β-CD did not exhibit any prominent sharp peaks, displaying only very broad peak at 18°, consistent with previous observations [26]. The lower crystallinity is attributable to packing disruption caused by hydroxypropyl and methyl groups present in HP-β-CD and ME-β-CD [27].

The diffraction pattern of HAL powder is consistent with the previous reports and indicates that this material is both pure and crystalline [24]. Notably, the diffraction data for HAL/α-CD and HAL/ME-β-CD inclusion complexes demonstrate a lack of HAL crystalline features, in agreement with the above-discussed DSC data.

### Planaria Experiments

Planaria, non-parasitic aquatic worms of the phylum Platyhelminthes, have traditionally been used as an animal model in biology due to their regeneration characteristics [28]. Furthermore, planaria have also been employed as a valuable tool in toxicological testing [29]. More recently, the versatility of planaria has extended into the domain of neuroscience research, establishing them as a remarkably useful animal model. Planaria possess a ganglion situated beneath its eyespots. This ganglion, known as the cerebral ganglia, comprises a bilobed cluster of nerve tissue and is regarded as the worm’s brain [30]. Several research groups evaluated the effects of various psychoactive drugs on planaria locomotion and behavior [31].

The planarian nervous system uses many of the neurotransmitters found in vertebrates, including humans [32] and HAL is an antipsychotic drug which can have an influence on the nervous system and change their behaviour as assessed by their mobility. In this study, planaria has been used to assess the impact of HAL and its formulations with cyclodextrins (CDs).

**Fig. 6:**
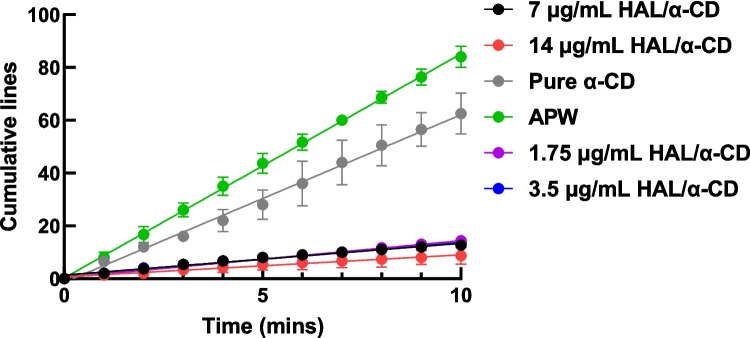
Representative linear regression of planaria movement over time.

**Fig. 7:**
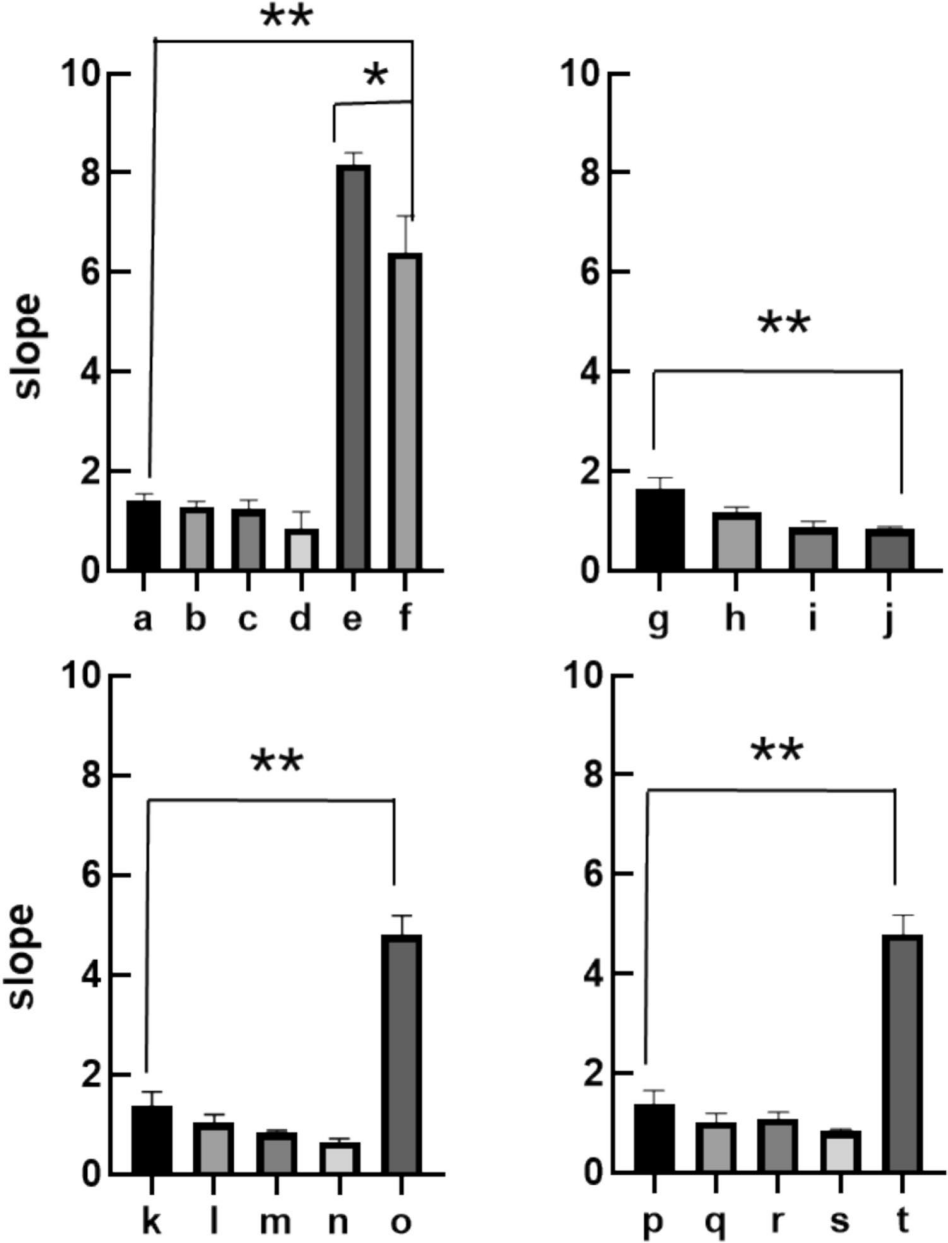
Planarian mobility slope values derived from linear regression of distance moved *versus* time in various sample solutions, including inclusion complexes of haloperidol (HAL) with different cyclodextrins (CDs), pure HAL, and CDs alone. The slope represents the distance moved (i.e., speed) by planaria over a 10-min period. The sample groups are as follows: (**a**) 1.75 μg/mL HAL/14 mg/mL α-CD; (**b**) 3.5 μg/mL HAL/14 mg/mL α-CD; (**c**) 7 μg/mL HAL/14 mg/mL α-CD;(**d**) 14 μg/mL HAL/14 mg/mL α-CD; (**e**) Artificial Pond Water (APW); (**f**) 14 mg/mL α-CD; (**g**) 1.75 μg/mL pure HAL; (**h**) 3.5 μg/mL pure HAL; (**i**) 7 μg/mL pure HAL; (**j**) 14 μg/mL pure HAL; (**k**) 1.75 μg/mL HAL/14 mg/mL ME-β-CD; (**l**) 3.5 μg/mL HAL/14 mg/mL ME-β-CD; (**m**) 7 μg/mL HAL/14 mg/mL ME-β-CD; (**n**) 14 μg/mL HAL/14 mg/mL ME-β-CD; (**o**) 14 mg/mL ME-β-CD; (**p**) 1.75 μg/mL HAL/14 mg/mL HP-β-CD; (**q**) 3.5 μg/mL HAL/14 mg/mL HP-β-CD; (**r**) 7 μg/mL HAL/14 mg/mL HP-β-CD; (**s**) 14 μg/mL HAL/14 mg/mL HP-β-CD; (**t**) 14 mg/mL HP-β-CD. * and ** indicate statistically significant differences with *p < 0.05 and **p < 0.01.

A straightforward experimental technique was employed to assess the mobility of planaria in solutions containing haloperidol, cyclodextrins, and their inclusion complexes. In each experiment, a single planarian is placed in a Petri dish filled with the solution of interest, which is then positioned on graph paper. The movement of the planarian is recorded on video, and the crossings of the graph paper grid are subsequently analyzed and presented graphically as cumulative line crossings over time. Then, the slope of each line was determined through linear regression (Fig. 6). These slopes were then utilized to construct a bar chart displaying the cumulative slopes (Fig. 7). By comparing the slopes, one can discern changes in mobility of planaria; an upward slope indicates better mobility with a greater speed. Pure HAL in solutions inhibited the mobility of planaria, and as the concentration of HAL increases, there is a progressively greater reduction in the mobility slopes, which suggests a dose-dependent relationship. This result is consistent with the pharmacological effects of haloperidol on higher animals, such as mammals [33]. For example, in rats, the administration of haloperidol induces catalepsy, characterized by temporary immobilization and restricted mobility. Similarly, haloperidol significantly limits the mobility of planaria flatworms in solution. This observation suggests that planaria may possibly serve as a lower animal model to substitute mammals in experiments on catalepsy.

Pure solutions of α-CD, HP-β-CD and ME-β-CD mildly restrained planarian mobility, resulting in reduction of the slope value from 8.20 ± 0.22 recorded in pure APW to 6.40 ± 0.73, 5.50 ± 0.06 and 4.80 ± 0.39, respectively. This reduction in planaria mobility was statistically significant (p < 0.05). However, there was no significant difference among the slopes recorded in the presence of HP-β-CD and Me-β-CD compared to APW (p > 0.05). Cyclodextrins are known in the literature to enhance drug permeability through the biological membranes [34]. However, in the planaria experiments, an unexpected trend emerged. Notably, pure HAL exhibited stronger mobility inhibition on planaria compared to HAL in CD inclusion complexes. This observation suggests that HAL in inclusion complexes faces greater difficulty in traversing the planarian epithelia, likely due to the strong complexation between HAL and CDs, which hinders drug release from the cavity. This finding is consistent with the *K*_st_ values obtained for the inclusion complexes of haloperidol and cyclodextrins, which confirm strong binding between the drug and the cyclodextrin, further supporting the observed effect.

## Conclusions

From the findings of the experiments outlined above, it is apparent that HP-β-CD, ME-β-CD, and α-CD offer substantial improvements in the solubility of unprotonated HAL within aqueous solutions. β-CD provided very modest improvements in the solubility of HAL, with a slight increase observed as the β-CD concentration rose to 8 mg/mL; in contrast, γ-CD did not produce any noticeable enhancements in the drug solubility. Notably, HP-β-CD and ME-β-CD emerged as the top performers, yielding nearly a 20-fold enhancement in HAL solubility. This underscores the potential of modified β-CD as a carrier for HAL, promising significant advancements in addressing its solubility challenges.

Moreover, the interaction between CD and HAL has been elucidated through various analytical techniques such as NMR, DSC, and Job’s plots, indicating a 1:1 stoichiometry. According to data from DSC and PXRD studies, haloperidol is presented in freeze-dried formulations of inclusion complexes in an amorphous state.

HAL in aqueous solutions exerts a strong effect on the mobility of planaria, resembling the catalepsy observed in mammalian models. This finding demonstrates the potential of planaria to replace mammals such as mice and rats in experimentation with haloperidol, its derivatives, analogues and their formulations. Using planaria in this context has important implications for 3Rs research, promoting the replacement of higher animals in experimentation and enhancing ethical standards in scientific research.

Despite HAL's ability to enter the CD cavity, as demonstrated by physicochemical analyses, the planaria study revealed that these CDs were unable to facilitate HAL's passage through the planarian membrane. This is likely attributed to the strong binding inside CDs cavity between the CDs and HAL, which has an inhibition of releasing HAL across the planaria’s membranes.

## Supplementary Information

Below is the link to the electronic supplementary material.

Supplementary file1 (DOCX 28 KB)

## Data Availability

The datasets generated in this study are available from the authors on reasonable request.
