# Supplementary material for: Cyclodextrin-mediated Enhancement of Haloperidol Solubility: Physicochemical Studies and In Vivo Investigation Using Planaria Worms
Source: Pharm Res. 2025 Aug 15;42(8):1373–83. doi: 10.1007/s11095-025-03909-0 (PMC12405015; doi:10.1007/s11095-025-03909-0)
Supplement: Supplementary file 1 — Supplementary file1 (DOCX 28 KB) [file 11095_2025_3909_MOESM1_ESM.docx]

Supplementary information to

**Cyclodextrin-mediated enhancement of haloperidol solubility: Physicochemical studies and *in vivo* investigation using planaria worms**

Yuehuai Xiong^1^, Kenneth Shankland^1^, Vitaliy V. Khutoryanskiy^1,2^*

*^1^Reading School of Pharmacy, University of Reading, Whiteknights, PO box 224, Reading RG66AD, United Kingdom*

*^2^Physicochemical, Ex Vivo and Invertebrate Tests and Analysis Centre (PEVITAC), University of Reading, Reading RG66AD, United Kingdom*


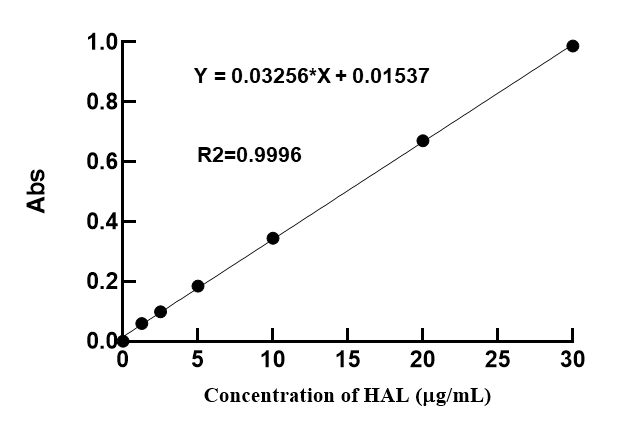


Supplementary figure 1. Calibration curve of HAL in UV. The R^2^ is 0.9996 that means the curve is nearly leaner. So it meets the requirements of calibration curve.
